# Examination of previously identified associations within the Genetic Analysis Workshop 19 data

**DOI:** 10.1186/s12919-016-0012-2

**Published:** 2016-10-18

**Authors:** Richard A. J. Howey, Jakris Eu-ahsunthornwattana, Rebecca Darlay, Heather J. Cordell

**Affiliations:** 1 Institute of Genetic Medicine, Newcastle University, Central Parkway, Newcastle Upon Tyne, NE1 3BZ UK; 2 Division of Medical Genetics, Department of Internal Medicine, Faculty of Medicine Ramathibodi Hospital, Mahidol University, Rama VI Rd, Ratchathevi, Bangkok 10400 Thailand

## Abstract

We investigate the possible replication of “known” associated single-nucleotide polymorphisms (SNPs) with blood pressure and expression phenotypes. Previous studies have provided a list of 95 SNPs thought to be associated with blood pressure phenotypes, of which 44 were present in the Genetic Analysis Workshop 19 (GAW19) family-imputed genome-wide association studies (GWAS) data and 4 in the GAW19 unrelateds sequence data. Using only the real (not simulated) GAW19 data, we show through the use of statistical tests that account for family relatedness, using FaST-LMM (Factored Spectrally Transformed Linear Mixed Model), that none of our candidate SNPs yields a significant *p* value. Furthermore, a study of epistasis, aiming to detect statistical interactions between loci with respect to their association with transcription levels has provided a list of 30 associated interacting SNP pairs, of which 13 are present in the GAW19 family GWAS and expression data. We show for this set of results, using the program GEMMA (genome-wide efficient mixed-model analysis) to account for family relatedness, that there is evidence of replication within the real GAW19 data. Two individual SNP pairs reach significance, and the set of remaining results give a combined *p* value of 0.017 that at least 1 of these remaining SNP pairs interacts to influence an expression phenotype.

## Background

Previous studies using very large data sets have provided a list of single-nucleotide polymorphisms (SNPs) believed to be associated with blood pressure and expression phenotypes. We attempt to replicate these SNPs in the Genetic Analysis Workshop 19 (GAW19) family genome-wide association studies (GWAS) data set and GAW19 sequence data, which may indicate the feasibility of finding novel SNPs in the GAW19 data sets.

## Methods

### Family genome-wide association studies data

The GAW19 family GWAS data [1] consisted of 959 individuals in 20 families with SNP data for odd chromosomes, including both real and imputed SNP data, and phenotype data for systolic blood pressure (SBP), diastolic blood pressure (DBP), and hypertension (HTN). Quality control was performed identically to that by Eu-ahsunthornwattana et al. [2] on the Genetic Analysis Workshop 18 data. This resulted in 954 individuals in 20 families. The phenotype data consists of longitudinal data measured over 4 years with covariates for smoking, HTN medication, and age. Covariates and measurements over multiple time points were accounted for by transformation to a single “average” quantitative trait for each phenotype, as described by Eu-ahsunthornwattana et al. [2].

A meta-analysis study conducted by Tragante et al. [3] of 87,736 individuals provided 95 candidate SNPs associated with blood pressure–related phenotypes; of these, 44 candidate SNPs were present in the GAW19 family data. Two extra phenotypes were created using the GAW19 phenotype data as defined by Tragante et al. [3]: (a) median arterial pressure (MAP) = 2/3 DBP + 1/3 SBP; and (b) pulse pressure (PP) = SBP − DBP.

The 44 candidate SNPs were tested individually using FaST-LMM (Factored Spectrally Transformed Linear Mixed Model) with the realized relationship matrix (RRM) option to adjust for relatedness between individuals. To examine the overall association of a set of SNPs we defined a statistic inspired by Dudbridge and Koeleman’s rank truncated product statistic [4]:

$$ -{\displaystyle {\sum}_{i=1}^n{ \log}_{10}}\kern0.5em {p}_i $$

where *p*_i_ is the *p* value of the *i* ^*th*^ SNP tested from *n* candidate SNPs. Candidate SNPs for each phenotype were considered together giving overall *p* values for DBP, MAP, PP, SBP, and HTN. The null hypothesis is formed by assuming that none of the SNPs are associated with the phenotype in question. A set of *p* values can be generated by sampling U[0, 1], where the correlations (*r* ^2^, calculated using PLINK) of nearby SNPs (<2 Mb) are accounted for [5]. The overall *p* value is then given by the proportion of simulated test statistics greater than the observed test statistic, from 500,000 replicates generated under the null hypothesis. An alternative method to control the false discovery rate for correlated test statistics is given by Yekutieli and Benjamini [6].

Estimates of the power to detect association, at significance level 0.05, for each of the tested SNPs with the appropriate phenotypes were calculated using the program Quanto (http://biostats.usc.edu/Quanto.html), assuming that the individuals are unrelated, thus providing upper limits for the power. Parameter estimates and minor allele frequencies were taken from Tragante et al. [3], and sample sizes were assumed to equal those of the GAW19 family GWAS data.

### Unrelated sequence data

From the 95 previously associated SNPs, only 5 were found in the GAW19 sequence data. Data consisted of 1943 unrelated individuals (of which 92 had missing phenotype data), together with 1 covariate on HTN medication. PLINK was used to calculate *p* values using linear regression.

### Expression data

A recent study by Hemani et al. [7], motivated by a desire to investigate the extent to which epistasis (the phenomenon whereby one polymorphism’s effect on a trait depends on other polymorphisms present in the genome) might influence complex traits, detected 30 gene–gene (SNP–SNP) interactions associated with transcription. We attempted to replicate these associations using the GAW19 family GWAS and expression data. From the 30 candidate SNP pairs, 11 were not considered because SNPs were on even chromosomes and 6 because of missing gene-probe data. The gene probes in the GAW19 data were different from those used by Hemani et al. [7], and were adjusted to account for covariates using the same method as was described for the blood pressure phenotypes. One gene *(CTSC)* had 2 gene probes.

To test for SNP–SNP interactions while allowing for family relatedness, GEMMA (genome-wide efficient mixed-model analysis) was used with an estimated kinship matrix. GEMMA does not have an interaction option but it does allow covariates, which were used to encode SNP data through use of 2 linear mixed models. The first model encoded 3 variables: the number of minor alleles for each SNP and the intercept. The second model encoded an extra variable given by the product of the number of minor alleles of the 2 SNPs, thus imposing an additive × additive interaction model. The maximum likelihood estimates for each model were used to evaluate *p* values using the likelihood ratio test.

An overall *p* value for all 14 interaction tests was calculated using the method previously described for single SNP association analyses in the GAW19 family GWAS data, using 10 million replicates.

## Results

### Family genome-wide association studies data

**Fig. 1:**
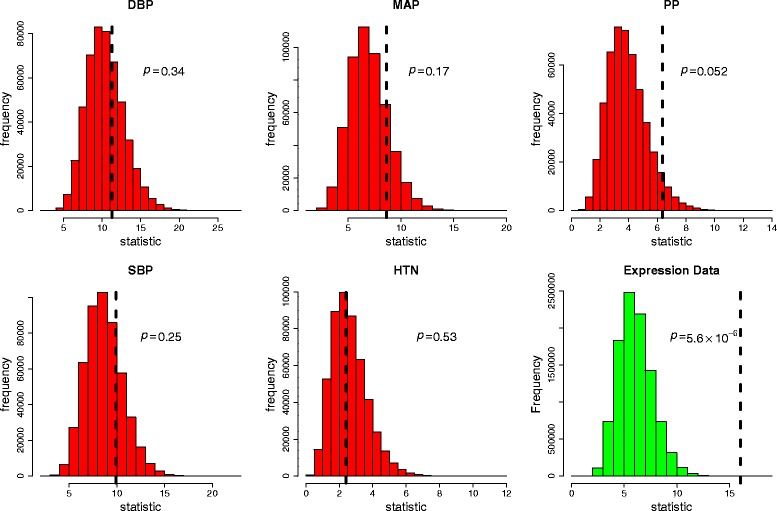
GWAS family data and expression data, permutation distributions. Permutation distributions for the GWAS and expression data *p* values with observed test statistics shown by the dashed lines

Table 1 lists the results for the SNP analyses using the imputed family GWAS data. It can be seen that no SNPs are below the Bonferroni corrected *p* value of 0.05/75 = 0.00067 for a family-wise error rate (FWER) of 0.05. Figure 1 shows the overall permutation distributions for the test statistics for each phenotype resulting in *p* values of 0.34, 0.17, 0.052, 0.25, and 0.53 for DBP, MAP, PP, SBP, and HTN respectively. This appears to provide some very weak evidence of association for PP; that is, at least 1 of the tested SNPs is significantly associated with PP.

**Table 1:** GWAS family data

SNP	Chr	Position	DBP	MAP	PP	SBP	HTN
rs880315	1	10796866	0.75			0.00902	
rs4846049	1	11850365	0.0971				
rs17367504	1	11862778	0.0729	0.0695		0.205	0.323
rs13306560	1	11866183		0.685			
rs5068	1	11905974	0.646				
rs17030613	1	113190807	0.967				
rs2932538	1	113216543	0.872				
rs2169137	1	204497913	0.276				
rs2004776	1	230848702				0.599	0.771
rs11122587	1	230867100	0.635				
rs347591	3	11290122				0.294	
rs13082711	3	27537909	0.674	0.527			
rs3774372	3	41877414	0.0539				
rs9815354	3	41912651	0.0539				
rs319690	3	47927484		0.795			
rs419076	3	169100886	0.708	0.549		0.519	
rs7726475	5	32575914	0.224			0.907	
rs1421811	5	32714270				0.577	
rs1173766	5	32804528	0.992				
rs1173771	5	32815028	0.913		0.266	0.36	0.857
rs11953630	5	157845402	0.273	0.445		0.86	
rs2282978	7	92264410			0.618		
rs17477177	7	106411858			0.00845		
rs3918226	7	150690176	0.502				
rs10224002	7	151415041				0.913	
rs661348	11	1905292		0.158			
rs217727	11	2016908		0.0255		0.0144	
rs7129220	11	10350538			0.0894		
rs2014408	11	16365282		0.604			
rs381815	11	16902268	0.945	0.794		0.643	
rs757081	11	17351683		0.299	0.0322	0.0537	
rs2074311	11	17421860			0.238		
rs3741378	11	65408937		0.599		0.352	
rs633185	11	100593538	0.0419	0.0564		0.218	0.289
rs11222084	11	130273230			0.504		
rs1036477	15	48914926			0.951		
rs1378942	15	75077367	0.629	0.875		0.774	0.126
rs6495122	15	75125645	0.145	0.0616			
rs2071410	15	91420940					0.517
rs2521501	15	91437388	0.523	0.46		0.455	
rs12946454	17	43208121				0.919	
rs17608766	17	45013271			0.943	0.936	
rs12940887	17	47402807	0.451			0.443	
rs16948048	17	47440466	0.338				

Replication *p* values in the GAW19 imputed family GWAS data for SNPs and phenotypes as indicated. Only previously associated SNP–phenotype combinations were investigated

Upper limits of the power to detect the tested SNP associations, at significance level 0.05, gave estimates ranging from approximately 0.080 to 0.30.

### Unrelateds sequence data

Table 2 lists the results for SNP analyses using sequence data. No SNPs are below the Bonferroni corrected *p* value (0.05/12 = 0.0042) for a FWER of 0.05.

**Table 2:** Unrelateds sequence SNP data

SNP	Chromosome	Position	DBP	MAP	PP	SBP
rs3774372	3	41877414	0.974			
rs661348	11	1905292		0.497		
rs217727	11	2016908		0.789		0.490
rs757081	11	17351683		0.375	0.375	0.432
rs3741378	11	65408937		0.942		0.772
rs2472304^a^	15	75044238	0.246	0.275		0.472

Replication *p* values in the GAW19 sequence data for SNPs and phenotypes as indicated. Only previously associated SNP-phenotype combinations were investigated

^a^Using the proxy SNP rs1378942

### Expression data

Table 3 lists the results for the SNP pair analyses using the GWAS and expression data. Two SNP pairs gave *p* values below the Bonferroni corrected *p* value of 0.0036 for a FWER of 0.05: rs4284750 and rs873870 on chromosome 19 interacted to influence gene expression at *ATP13A1;* and rs9979356 and rs3761385 on chromosome 21 interacted to influence gene expression at *CSTB*. The *p* values are generally lower than expected with a median of 0.223. Furthermore, the overall *p* value for the 14 tests, as shown in Fig. 1, is 5.6 × 10^−6^, and with the 2 significant SNP pairs removed the remaining 12 tests give an overall *p* value of 0.017, which provides reasonable evidence that at least 1 of these SNP pairs also interacts to influence the corresponding gene expression measurement.

**Table 3:** Gene expression data and SNP–SNP interactions

Gene	Probe	SNP 1	Chr 1	SNP 2	Chr 2	*p* value
*ATP13A1*	GI_9966896-S	rs4284750	19	rs873870	19	0.000101
*CSTB*	GI_20357564-S	rs9979356	21	rs3761385	21	0.000747
*CTSC*	GI_22538439-I	rs7930237	11	rs556895	11	0.020446
*CTSC*	GI_22538438-I	rs7930237	11	rs556895	11	0.636009
*FN3KRP*	GI_20149679-S	rs898095	17	rs9892064	17	0.015972
*GAA*	GI_11496988-S	rs11150847	17	rs12602462	17	0.240101
*LAX1*	GI_8923315-S	rs1891432	1	rs10900520	1	0.205055
*MBNL1*	GI_41281590-S	rs16864367	3	rs13079208	3	0.01683
*MBNL1*	GI_41281590-S	rs7710738	5	rs13069559	3	0.938258
*MBNL1*	GI_41281590-S	rs218671	17	rs13069559	3	0.178989
*MBNL1*	GI_41281590-S	rs11981513	7	rs13069559	3	0.307821
*PRMT2*	GI_4504494-S	rs2839372	21	rs11701058	21	0.4209
*TRA2A*	GI_33620726-S	rs7776572	7	rs11770192	7	0.51894
*VASP*	GI_4507868-S	rs1264226	19	rs2276470	19	0.645204

Replication *p* values in the GAW19 GWAS data for interacting SNP pairs and gene probes as indicated

## Discussion

The candidate SNPs and blood pressure phenotypes investigated here were previously detected in large meta-analyses or other replicated studies, giving considerable confidence that these SNPs are in fact genuinely associated. However, the sample size in the GAW19 family GWAS data consists of only 954 related individuals, giving power (for nominal *p* value 0.05) expected to be less than 0.080 to 0.30; perhaps it is not too unexpected that no associations were replicated. The sample size of the unrelateds sequence data, 1943 individuals, was greater, but nonetheless did not replicate any previously observed associations. Although the low sample sizes are the most obvious reason for the nonreplication, there may also be more subtle reasons for the nonreplication, such as the relatedness and ethnicity of the samples used. The quality and accuracy of the measured phenotypes may also be relevant, in particular whether individuals took HTN medicine or not.

The SNP–SNP interactions previously shown to be associated with transcription did, however, show some evidence of replication, with 2 SNP pairs showing significant evidence of association and the remaining SNP pairs giving an overall *p* value of 0.017, indicating that at least 1 additional SNP pair is associated. It is argued that the power to detect such associations may be greater because of the more direct link between SNPs and transcription. We note, however, that the interpretation of such findings as representing genuine interactions (as opposed to haplotype effects, possibly marking an untyped causal variant) can be flawed when the SNPs are close to one another [8].

## Conclusions

There was no evidence of replication using the GAW19 data for previously found SNP associations with blood pressure phenotypes, possibly because of the low sample size. However, there was some evidence of replication for SNP–SNP interactions associated with transcription. This may be the result of a greater power to detect associations with transcription than with more distantly related phenotypes.
